# Essential headaches in developmental age: What is changed before, during and after the lockdown for COVID-19 pandemic. Clinical study

**DOI:** 10.3389/fped.2023.1166984

**Published:** 2023-04-25

**Authors:** Alice Bonuccelli, Greta Depietri, Tommaso Baldaccini, Irene Ricciutelli, Diego Peroni, Alberto Spalice, Gabriele Massimetti, Riccardo Morganti, Alessandro Orsini, Pasquale Striano

**Affiliations:** ^1^ PediatricNeurology, Pediatric University Department, Azienda Ospedaliero Universitaria Pisana, University of Pisa, Pisa, Italy; ^2^ Pediatric Clinic, Pediatric University Department, Azienda Ospedaliero Universitaria Pisana, University of Pisa, Pisa, Italy; ^3^ Department of Pediatrics, Child Neurology Division, Sapienza University Rome, Roma, Italy; ^4^ Psychiatric Clinic, Department of Clinical and Experimental Medicine, University of Pisa, Pisa, Italy; ^5^ Division of Statistical Support to Clinical Studies, Department of Clinical and Experimental Medicine, University of Pisa, Pisa, Italy; ^6^ “IRCCS Istituto Giannina Gaslini” member of ERN-Epicare, Genova, Italy; ^7^ Department of Neurosciences, Rehabilitation, Ophthalmology, Genetics, Maternal and Child Health, University of Genova, Genova, Italy

**Keywords:** headaches, COVID-19, lockDown, pandemic, video-terminal

## Abstract

**Introduction:**

Essential headache is one of the main causes of pain in children, and has an important impact on their quality of life. In children with essential headaches play an important role in both triggers like stress, excessive use of video terminals, or physical fatigue but also comorbidities like anxiety, depression, and sleep disturbances. CoViD-19 Pandemic was very stressful, especially for children, and amplified all headache triggers and comorbidities.

**Study objective:**

In this work, we studied the aspects concerning the headache,lifestyle, habits, and mental health of children before, during, and after the lockdown and the differences between some categories (selected by age, gender, and headache status before the lockdown).

**Methods:**

This study was conducted on 90 patients with primary headaches followed at the AOUP Neuropediatrics Clinic from January 2018 to March 2022. Participants answered a questionnaire of 21 questions. For every question, the answer was divided into three periods: before, during, or after the lockdown. All dates have been converted and inserted into a database and we used SPSS technology for statistical analysis.

**Results:**

In our study, 51,1% were females and 48,9% were males and there was a prevalence of adolescents (56,7%) compared to children from 5 to 11 years (43,3%). Regarding the headache onset, 77,7% of patients started to suffer from headaches before 10 years, moreover, 68,9% had familiarity with the headache. Using Cohen's K- Concordance Test, we performed a Concordance Analysis, studying the questions in the three periods above mentioned: considering headache characteristics there is poor concordance about the trend of headache; modest concordance (K: 0,2–0,4) about the frequency and the type (migraine or tension headache); moderate concordance (K: 0,41–0,61) about the acute use of analgesic. Analyzing lifestyle the lockdown had a significant impact on sports (practiced much less) and on the use of video terminals (used much more).

**Conclusion:**

The pandemic and lockdown aren't events that led to strong and unidirectional responses in patients, there is great variability in the answers about headache, lifestyle, and psychology, and each patient had individualized reactions. However, these considerations are not applied to physical activity and the use of video terminals, because both have been inevitably modified by the pandemic situations and so were not affected by subjective influence.

## Introduction

Headache is defined as a limited or diffuse, continuous or sporadic painful sensation affecting the head. The International Classification of Headache Disorders (ICHD), now in its 3rd edition, divided headaches into three principal categories: Primary headaches, Secondary Headaches, Painful cranial neuropathies, and facial pain (1). Primary Headache (PH) is a paroxysmal multifactorial and complex disorder, characterized by the recurrence of symptoms interspersed with physical and mental well-being (2). ICHD-3 divides Primary Headaches into four principal types: Migraine, Tension-type headache, Trigeminal autonomic cephalalgia, and Other primary headache disorders (1).

The diagnosis is clinical, based on accurate anamnesis and the general and neurological physical examination (3, 4). It's one of the three main causes of pediatric pain after muscular-skeletal and abdominal pain. Primary Headache is also the first reason for absenteeism from school and the most frequent neurological cause of access to the pediatric emergency room. Due to her debilitating nature and the repercussions on the family and school, headache has a significant social impact in this life period.

The prevalence of Primary headaches in childhood varies from 5.9% to 82%, increasing progressively with age and reaching a peak around 13 years (5). Moreover, the prevalence of headaches increases more in girls (ratio of 3: 1) during puberty (6). Specific triggers such as stress, sleep deprivation, fever, loud noises, starvation, abuse of technological devices, etc. can cause or aggravate headache episodes going to act as a stimulus for the activation of pathogenetic mechanisms. Recognizing and preventing them is essential to reduce the frequency of attacks and improve the patient's quality of life (Table 1). Avoiding these triggers should reduce their frequency and improve the patient's quality of life (7–9). The Covid-19 pandemic has been a stressful event that has disrupted everyone's life with different psychological reactions. this event can be taken as an example to understand how stressful and psychological factors can influence headaches in a specific age group (10). In particular, regarding childhood primary headaches, some studies have evaluated the short and long-term effects of the lockdown; the short-term effects seem to be beneficial for headaches and patients improve activating coping mechanisms (11, 12).

**Table 1:** Principal risk factors.

PRINCIPAL RISK FACTORS
Psychological factors: stress, emotions, mood changes
Physical fatigue
Excessive use of video-terminals
Closed places
Bright light, loud noises and smells
Smoke
Weather variations
Fever
Hormonal factors
Excessive sleep or deprivation
Foods and beverages containing nitrates, glutamate, tyramines

In the long term, patients report a worsening of headaches, related to sleep disturbances, dietary changes, increased stress, anxiety, and depression, with the potential to evolve into a chronic form. This information clarifies how lifestyle changes can affect headaches (13, 14).

**The objective of the study:** This is a retrospective study to value the clinical differences of primary headaches in a pediatric population concerning the Covid-19 pandemic.

The primary outcomes of this study were to evaluate the variations of primary headache frequency and medication use in three periods: before, during, and after lockdown; furthermore, if these changes are statistically correlated with lifestyle changes due to restrictive measures of the Covid-19 emergency. A secondary outcome was to value the differences in these parameters in school-age children and adolescents.

## Materials and methods

### Population

We included n° 90 patients from 5 to 18 years old with a diagnosis of headache (migraine, migraine with aura, TTH) followed in Neuropediatric of the University Hospital of Pisa from January 2018 to March 2022.

The patients were divided into different categories based on: (1) Age: children (5–11 years old) and adolescents (12–18 years old); (2) Sex: female and male; (3) School attended: elementary school, middle school or high school; (4) Age of the primary headache onset: before 10 years old and after 10 years old; (5) Familiarity: present or absent.

### Analysis tools

A questionnaire with 21 questions, made *ad hoc* by the authors, was sent by e-mail. All the 90 patients included in the study completed the questionnaire, responding to all questions. Parents helped children (5–8 years) answer the questionnaire, reading the questions together and evaluating the best answer together.

**Figure 1:**
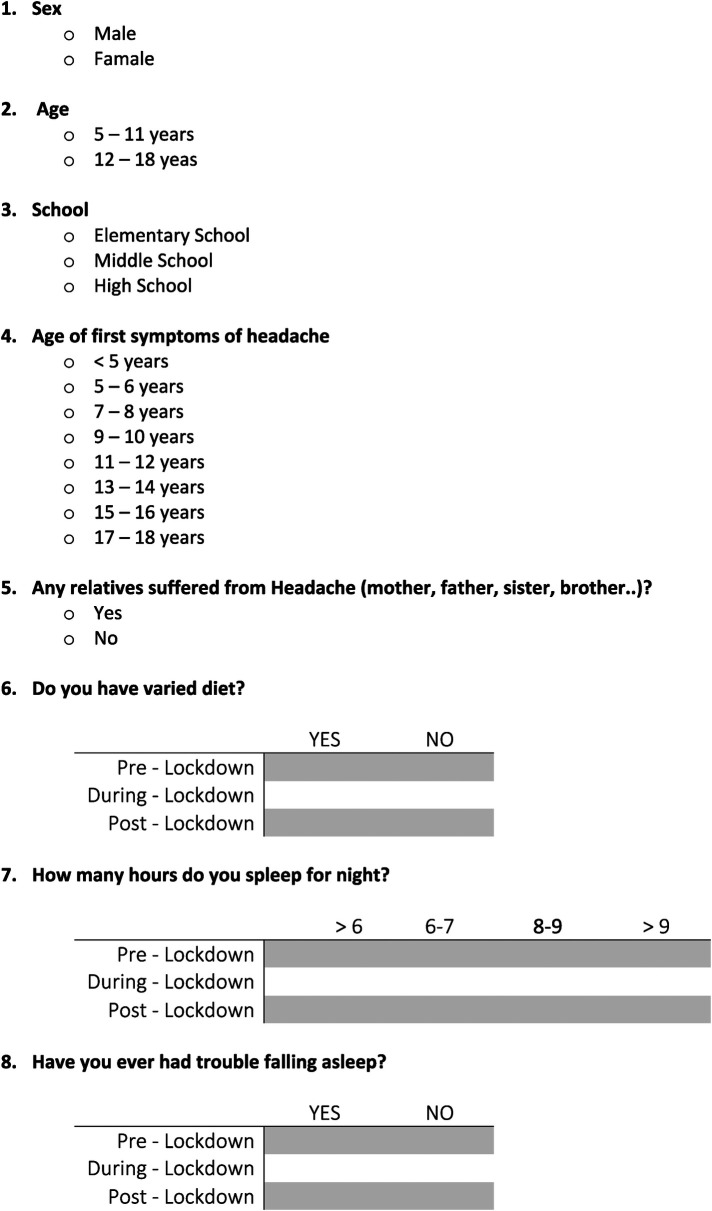
Questionnaire made ad hoc.

The first three questions related to demographic data: age, sex, and school attended. The next two inquired about the age of onset of primary headache and familiarity. The last 16 questions investigated different elements: characteristics of the primary headache, lifestyle, and psychological status of the patients. Each question was asked for the three periods: before, during, or after the lockdown. All patients signed informed consent to the study (Figure 1).

### Statistical analysis

Categorical data were described by absolute and relative (%) frequency. To analyze the concordance between categorical repeated measures Cohen's test was performed. All analyzes were carried out with SPSS v.28 technology (Table 2).

**Table 2:** Kappa value in Cohen's test.

Kappa Value	Agreement
<0,2	Poor
0,2–04	Modest
0,41–0,61	Moderate
0,61–0,80	Good
>0,80	Excellent

## Results

### Population characteristics

In our study 51.1% of the population was female. Regarding age, we found a prevalence of adolescents (56.7%) compared to children aged 5 to 11 (43.3%). 40% of patients attended Primary school, 38,9% Middle school, and 21,1% High School. The children with primary headache onset before 10 years were 77.7%, and those with onset after 10 years were 22.3%. Finally, 68.9% of patients had at least one family member who suffers or had suffered from a headache (Table 3).

**Table 3:** Population characteristics.

Parameters	Frequency	Percentage
Sex		
F	46	51,1
M	44	48,9
Age		
5–11	39	43,3
12–18	51	56,7
School		
Primary	36	40,0
Middle	35	38,9
High	19	21,1
Headache Onset		
Before 10 years	70	77,7
After 10 years	20	22,3
Familiarity		
No	28	31,1
yes	62	68,9

### Concordance analysis

#### Headache

We found a good concordance for migraine with or without aura or tension-type headaches in the three periods. Children with migraine were 42.2% pre-lookdown, 36.7% during the lockdown, and 40% post-lookdown. We concluded that there wasn't a significant variation in the type of primary headache in the three periods and tensive-type headache remained the most widespread type of headache (Table 4).

**Table 4:** Concordance analysis about headache characteristics.

HEADACHE				
	Frequency	Percentage	Agreement	
Type of headache pre	** **		Pre vs. During	Pre vs. Post
Migraine	38	42,2	Good	Good
Tension headache	52	57,8		
Type of headache during	** **		During vs. Post	
Migraine	33	36,7	Good	
Tension headache	57	63,3		
Type of headache post				
Migraine	36	40,0		
Tension headache	54	60,0		
Frequency days/month pre	** **		Pre vs. During	Pre vs. Post
0	17	18,9	Modest	Modest
1–3	22	24,4		
4–7	25	27,8		
8–10	18	20,0		
11–14	4	4,4		
More than 15	4	4,4		
Frequency days/month during	** **		During vs. Post	
0	18	20,0	Modest	
1–3	26	28,9		
4–7	24	26,7		
8–10	13	14,4		
11–14	3	3,3		
More than 15	6	6,7		
Frequency days/month post	** **			
0	7	7,8		
1–3	29	32,2		
4–7	23	25,6		
8–10	17	18,9		
11–14	5	5,6		
More than 15	9	10,0		

Regarding the frequency of primary headaches, we have identified six categories (shown in Table 2), without finding statistically significant differences before, during, and after the lockdown. The biggest difference regards the category of those who have never had a headache in the two periods “during and post-lockdown”. The percentage of this response goes from 20% to 7.8%, highlighting a trend of increasing headache attendance when activities resume. We supposed that the cause could be attributable to the increased environmental demands that were triggered in some subjects.

#### Drugs

We analyzed the use of drugs for primary headache both in acute, during headache attack, and for prophylaxis, to prevent the exacerbation; we found a moderate concordance between the periods “pre” and “during” lockdown and a modest concordance comparing “pre” and “post” lockdown or “during” and “post” lockdown. Furthermore, in the questionnaire, we asked those who used prophylactic drugs to list the type of drug used. We also evaluated whether the lookdown had increased the need for psychotherapy but the concordance between the three periods was moderate. 10 patients used psychotherapy before the lockdown and only 2 were added in the post-period. These 10 patients didn't have any certified psychiatric pathology (Table 5).

**Table 5:** Concordance analysis about drugs.

DRUGS				
	Frequency	Percentage	Agreement	
Acute drugs pre			Pre vs. During	Pre vs. Post
Never	35	38,9	Moderate	Modest
1–3	30	33,3		
4–7	15	16,7		
8–10	8	8,9		
More than 10	2	2,2		
Acute drugs during			During vs. Post	
Never	36	40,0	Modest	
1–3	35	38,9		
4–7	12	13,3		
8–10	5	5,6		
More than 10	2	2,2		
Acute drugs post				
Never	24	26,7		
1–3	40	44,4		
4–7	10	11,1		
8–10	11	12,2		
More than 10	5	5,6		
Prophylaxis drugs pre			Pre vs. During	Pre vs. Post
No	67	74,4	Moderate	Modest
Yes	23	25,6		
Prophylaxis drugs during			During vs. Post	
No	67	74,4	Modest	
Yes	23	25,6		
Prophylaxis drugs post				
No	60	66,7		
Yes	30	33,3		
Psychotherapy pre			Pre vs. During	Pre vs. Post
No	80	88,9	Moderate	Moderate
Yes	10	11,1		
Psychotherapy during			Moderate	
No	85	94,4		
Yes	5	5,6		
Psychotherapy post				
No	78	86,7		
Yes	12	13,3		

#### Quality life

We found excellent concordance between the periods “pre” and “during” lockdown and “pre” and “post” lockdown and good concordance comparing the periods “during” and “post” lockdown. There was a trend towards a less healthy diet during the lockdown, but the change was not significant (Table 6).

**Table 6:** Concordance analysis about Quality Life.

**QUALITY LIFE**				
	Frequency	Percentage	Agreement	
**Healty diet pre**	** **		Pre vs During	Pre vs Post
no	20	22,2	Excellent	Excellent
yes	70	77,8		
**Healty diet during**	** **		During vs Post	
no	26	28,9	Good	
yes	64	71,1		
**Healty diet post**	** **			
no	22	24,4		
yes	68	75,6		
**Hours of sleep pre**	** **		Pre vs During	Pre vs Post
<=7	16	17,8	Good	Moderate
>7	74	88,2		
**Hours of sleep during**	** **		During vs Post	
<=7	17	18,8	Moderate	
>7	73	81,2		
**Hours of sleep post**	** **			
<=7	27	30,0		
>7	63	70,0		
**Sleep disorders pre**	** **		Pre vs During	Pre vs Post
no	73	81,1	Modest	
yes	17	18,9		
**Sleep disorders during**	** **		During vs Post	
no	67	74,4	Moderate	
yes	23	25,6		
**Sleep disorders post**	** **			
no	67	74,4		
yes	23	25,6		
**Physical activity pre**	** **		Pre vs During	Pre vs Post
no	18	20,0	Poor	Modest
yes	72	80,0		
**Physical activity during**	** **		During vs Post	
no	68	75,6	Poor	
yes	22	24,4		
**Physical activity post**	** **			
no	24	26,7		
yes	66	73,3		
**Exposure video terminals hours/day pre**	** **		Pre vs During	Pre vs Post
1-3	70	77,8	Poor	Poor
4-6	17	18,9		
7-9	3	3,3		
**Exposure video terminals hours/day during**	** **		During vs Post	
1-3	15	16,7	Poor	
4-6	42	46,7		
7-9	21	23,3		
More than 10	12	13,3		
**Exposure video terminals hours/day post**	** **			
1-3	47	52,2		
4-6	33	36,7		
7-9	8	8,9		
More than 10	2	2,2		

The hours of sleep were distinguished in less or more than 7 h per night and we found a good concordance between “pre” and “during” lockdown, while a moderate concordance was found between “pre” and “post” lockdown or “ during” and “post” lockdown. Overall, there is a progressive increase in patients who sleep less, going from 17.8% to 30% in the activity recovery phase.

Regarding the quality of sleep, we asked if the patients had difficulty falling asleep or if they were subject to nocturnal awakenings. From “pre” to “during” lockdown, patients with sleep problems increased from 18.9% to 25.6% and this situation remained unchanged even in the “post”. About video terminals, there was a significant increase in hours of exposure during the lockdown, attributable both to online lessons and to the absence of alternative activities. We divided the exposure to video terminals into four categories: 1–3 h a day, 4–6 h a day, 7–9 h per day, and more than 10 h a day. Patients who used electronic devices for 7–9 h per day increased from 3,3% in “pre” lockdown to 13,3% during the lockdown and decreased again to 2,2% in post-lockdown.

Finally, we stratified the subjects according to gender (males and females) and age group (5–11 and 12–18) and performed a stratified analysis considering the responses given by the patients to the question on headache self-rating. The variability in this response is greater in females and adolescents, probably due to the different sensitivity of females and adolescents towards an event such as leading to a radical change in habits and sociability.

## Discussion

Data analysis has highlighted some significant aspects of headaches in the periods evaluated. First, the large variability of many of the categories analyzed in the absence of a unidirectional trend.

In absolute terms, in the post-lockdown period, there was a slightly worsening trend in headaches compared to the pre-lockdown period, although in the absence of statistical significance, there are some subjects whose headaches improved during the lockdown period and others whose headaches got worse, according to the study by Dellavalle G. et al.l (15). The cases which presented a worsening headache go from 22.2% to 35.6%, compared to subjects who have an improvement in headache, who instead increased from 31.1% to 35.6%, even if there is no statistical significance. Probably the first group is subjects who suffered from a situation of environmental stress (school, sport, friendships, etc.), which failed with the lockdown. Conversely, the second group probably suffered more than the others from the discomfort caused by the restrictions resulting in the worsening of their headaches.

Considering the specific categories, adolescents and women were more affected by the influence of the lockdown, probably due to a greater sensitivity of these categories to the changes induced by the pandemic period (16, 17).

The only aspects that have undergone a statistically significant change are physical activity and hours of use of video terminals (18).

For the other lifestyle elements examined, such as nutrition, sleep, etc. no statistically significant changes were found (19, 20).

From all this, it can be concluded that the pandemic cannot be considered an event that induces a one-way and unambiguous variation toward the worsening of the headache. There are subjective factors that influence the response variably even in the presence of the same environmental stress factor.

Another interesting element that emerges is the failure to return to pre-pandemic values of headaches in the period following the lockdown. We can hypothesize that the population examined not only felt the effects of an extreme condition such as the lockdown but also that the psychological and social consequences persist even after the end of the lockdown.

## Conclusions

The Covid-19 pandemic was a dramatic event that radically changed people's lifestyles. In this context of sudden and prolonged restrictions, we have evaluated how sudden changes in routine habits ve affected the course of headaches.

Although lifestyle changes were common for all, there is not a single direction from the course of the headaches. The answers provided in the questionnaire are characterized by wide variability and only a slight variation in absolute value can be observed.

The pre-pandemic state of the headache also depended on the context so the improvement or worsening during the lockdown varied according to the presence of other stress and subjective psychological factors. The only exceptions are physical activity and abuse of video terminals which can be taken as examples of aspects whose variation is unidirectional and guided by the circumstance of confinement and not by the subjective response.

What emerges is the importance of evaluating the patient in all its complexity, since the treatment of any comorbidities, such as psychological problems and the correction of some lifestyles, are essential to obtain an appropriate clinical response.

## Data Availability

The original contributions presented in the study are included in the article/**Supplementary Material**, further inquiries can be directed to the corresponding author.
