# Supplementary material for: Essential headaches in developmental age: What is changed before, during and after the lockdown for COVID-19 pandemic. Clinical study
Source: Front Pediatr. 2023 Apr 25;11:1166984. doi: 10.3389/fped.2023.1166984 (PMC10168180; doi:10.3389/fped.2023.1166984)
Supplement: Supplementary file 1 [file Datasheet1.pdf]

1. **Sex**
   - Male
   - Famale

2. **Age**
   - 5 – 11 years
   - 12 – 18 yeas

3. **School**
   - Elementary School
   - Middle School
   - High School

4. **Age of first symptoms of headache**
   - < 5 years
   - 5 – 6 years
   - 7 – 8 years
   - 9 – 10 years
   - 11 – 12 years
   - 13 – 14 years
   - 15 – 16 years
   - 17 – 18 years

5. **Any relatives suffered from Headache (mother, father, sister, brother..)?**
   - Yes
   - No

6. **Do you have varied diet?**

	YES	NO
Pre - Lockdown	☐	☐
During - Lockdown	☐	☐
Post - Lockdown	☐	☐

7. **How many hours do you spleep for night?**

	> 6	6-7	8-9	> 9
Pre - Lockdown	☐	☐	☐	☐
During - Lockdown	☐	☐	☐	☐
Post - Lockdown	☐	☐	☐	☐

8. **Have you ever had trouble falling asleep?**

	YES	NO
Pre - Lockdown	☐	☐
During - Lockdown	☐	☐
Post - Lockdown	☐	☐

9. Do you play sports?

	YES	NO
Pre - Lockdown	☐	☐
During - Lockdown	☐	☐
Post - Lockdown	☐	☐

10. What type of headache do you have?

	Throbbing pain	Oppressive pain
Pre - Lockdown	☐	☐
During - Lockdown	☐	☐
Post - Lockdown	☐	☐

11. How many days a month have you headache?

	never	1-3	4-7	8-10	11-14	>15
Pre - Lockdown	☐	☐	☐	☐	☐	☐
During - Lockdown	☐	☐	☐	☐	☐	☐
Post - Lockdown	☐	☐	☐	☐	☐	☐

12. Pre – Lockdown how many times have you these moods?

	Never	Few times	Often	Always
Nervous, anxious	☐	☐	☐	☐
Worried	☐	☐	☐	☐
Restless	☐	☐	☐	☐
Unable to sit still	☐	☐	☐	☐
Jittery	☐	☐	☐	☐
Apathetic	☐	☐	☐	☐
Sad	☐	☐	☐	☐
Tired	☐	☐	☐	☐
Poor Appetite	☐	☐	☐	☐
Distracted	☐	☐	☐	☐

13. During – Lockdown how many times have you these moods?

	Never	Few times	Often	Always
Nervous, anxious	☐	☐	☐	☐
Worried	☐	☐	☐	☐
Restless	☐	☐	☐	☐
Unable to sit still	☐	☐	☐	☐
Jittery	☐	☐	☐	☐
Apathetic	☐	☐	☐	☐

Sad	☐	☐	☐	☐
Tired	☐	☐	☐	☐
Poor Appetite	☐	☐	☐	☐
Distracted	☐	☐	☐	☐

14. Now how many times have you these moods?

	Never	Few times	Often	Always
Nervous, anxious	☐	☐	☐	☐
Worried	☐	☐	☐	☐
Restless	☐	☐	☐	☐
Unable to sit still	☐	☐	☐	☐
Jittery	☐	☐	☐	☐
Apathetic	☐	☐	☐	☐
Sad	☐	☐	☐	☐
Tired	☐	☐	☐	☐
Poor Appetite	☐	☐	☐	☐
Distracted	☐	☐	☐	☐

15. How many hours a day do you use video-terminals (telephone, pc, tablet, tv)?

	1-3	4-6	7-9	> 10
Pre - Lockdown	☐	☐	☐	☐
During - Lockdown	☐	☐	☐	☐
Post - Lockdown	☐	☐	☐	☐

16. In your opinion after the lockdown the school commitment is:

- Increased
- The same
- Reduced

17. How many times a month do you use drugs for headache?

	never	1-3	4-7	8-10	>10
Pre - Lockdown	☐	☐	☐	☐	☐
During - Lockdown	☐	☐	☐	☐	☐
Post - Lockdown	☐	☐	☐	☐	☐

18. Do you do psycoterapy?

	YES	NO
Pre - Lockdown	☐	☐
During - Lockdown	☐	☐
Post - Lockdown	☐	☐

19. Do you use drugs to prevent headache?

	YES	NO
Pre - Lockdown	☐	☐
During - Lockdown	☐	☐
Post - Lockdown	☐	☐

20. If your answer is YES, what drugs do you use?

____

21. In general, what do you think about your headache?

	Improved	Stable	Worsened
Pre - Lockdown	☐	☐	☐
During - Lockdown	☐	☐	☐
Post - Lockdown	☐	☐	☐
